# Anthocyanins, Microbiome and Health Benefits in Aging

**DOI:** 10.3390/molecules26030537

**Published:** 2021-01-21

**Authors:** Rachel Hair, Junichi R. Sakaki, Ock K. Chun

**Affiliations:** Department of Nutritional Sciences, University of Connecticut, Storrs, CT 06269, USA; rachel.hair@uconn.edu (R.H.); junichi.sakaki@uconn.edu (J.R.S.)

**Keywords:** anthocyanins, inflammation, oxidative stress, gut microbiome, bone loss, cardiovascular disease, cancer, neurodegenerative disorder

## Abstract

The percentage of individuals over the age of 60 is projected to reach 22% by 2050; chronic diseases associated with aging can present challenges for these individuals. Anthocyanins and the gut microbiome have each been studied as independent influencers of health. Both these factors have shown to have a positive effect on cardiovascular and bone health in individuals, as well as on the prevention or treatment of certain forms of cancers. Anthocyanins have shown to modulate the composition of the gut microbiome and may have overlapping mechanisms in the prevention and treatment of cardiovascular disease, cancer, neurodegenerative disorders and aging-associated bone loss. These health outcomes are responsible for the hospitalization and deaths of millions of Americans every year and they cost the United States billions of dollars each year to maintain, prevent and treat. Alternative methods of treatment and prevention are desired since conventional methods (surgical and pharmacological methods, physical therapy, etc.) can be costly and have significant side effects; evidence suggests that anthocyanins and the gut microbiome may be potential avenues for this. This review evaluates the findings of existing literature on the role of anthocyanins and the gut microbiome on health and their potential as a natural therapeutic agent or a target organ to provide an alternative to the conventional methods of disease prevention and treatment.

## 1. Introduction

By 2050, it is projected that the world’s population aged 60 years old and above will increase to 2 billion people, comprising approximately 22% of the total population [1]. The rate of aging can be somewhat controlled through various pathways, including genetic pathways and biochemical processes [2]. Aging is a natural, time-dependent, physiological process that occurs in individuals, resulting in an overall functional decline [2]. That functional decline can result in the development of aging-associated chronic diseases, including cardiovascular disease (CVD), cancer, neurodegenerative disorders and osteoporosis. CVD and cancer are the leading causes of death in older adults, while hypertension, a risk factor for CVD development, is the most common chronic disease in the same population [3]. Alzheimer’s disease (AD) is a progressive neurological disorder which has been identified as one of the most aging-linked diseases and is the leading cause of dementia [4]. Postmenopausal osteoporosis is of particular concern for older women, affecting one in four women 65 years of age and above [5]. 

Anthocyanins are found naturally in an abundance of dietary sources [6] and have been noted for their possible health benefits [7]. Anthocyanins are responsible for the pigment in many plant foods [8] and are copious in many berry varieties, including blackberries, blueberries and cranberries [9]. Their color will change depending on the pH of the food matrix; they can appear in foods as purple, red, or blue [10]. Pelargonidins, cyanidins, delphinidins, peonidins, petunidins and malvidins are the common classes of anthocyanins occurring naturally in food [11]. Anthocyanins are a class of flavonoid and contain a phenolic structure that contributes to their biological effects [10]. The bioavailability of anthocyanins has been debated, with current evidence suggesting anthocyanins are relatively more bioavailable than previously thought [6,12]. Nevertheless, researchers have documented positive health outcomes attributed to anthocyanins, including improved vascular function, cancer prevention [13] and bone health [14]. It has been suggested that the anti-inflammatory and antioxidative effects of anthocyanins contribute to their ability to prevent or delay the onset of certain adverse health conditions. In terms of their effect on inflammation, phenolic compounds have the ability to stop pro-inflammatory mediators; they do this by either blocking their production or their action [15]. Since anthocyanins are found naturally in dietary sources, their use in the prevention and treatment of adverse health events is of interest; anthocyanins could present a safe and inexpensive method for disease prevention with minimal side effects [16].

Anthocyanin-rich foods may influence the composition of the gut microbiome and act as a mediator for the positive health outcomes associated with anthocyanins. Anthocyanins can be digested by various structures in the gut to form metabolites that are transferred throughout the body and have positive biological effects [17,18]. The changes in the microbiome may be from other components of the food item, with some literature suggesting that the fiber content of the anthocyanin-rich food is responsible for the alterations made in the gut [19]. However, the metabolism of prominent anthocyanins have been documented to have a positive effect on overall gut integrity by reducing inflammation and oxidative stress [11]. Microorganisms residing in the gastrointestinal (GI) tract are collectively known as the gut microbiome [20]. The gut microbiome functions as an endocrine organ and is an integral part of food digestion, with two major catabolic pathways related to the breakdown of food [21]. The first pathway involves breaking down carbohydrates and producing short chain fatty acid (SCFA) metabolites [21]. The second pathway is responsible for the fermentation of proteins and also results in the production of SCFAs, as well as the following potentially toxic cometabolites: ammonia, amines, thiols, phenols and indoles [22]. Interindividual variations in gut microbiome composition are common [23] and there is evidence from both human and animal models that diet can have an impact on the diversity of the microbiome [19,24]. While these changes can occur rapidly, the effects are temporary [25] unless continuous stimulus is applied to maintain the shift in diversity [26]. This could include lifestyle changes of individuals, like strictly adhering to the Mediterranean diet which has been shown to positively influence gut microbiota [27].

The gut microbiome can provide benefits to its host, as it may even decrease the toxicity of cancer treatments [28] and play a key role in the immunotherapy of cancer [29]. The gut microbiota-host interaction has been studied as a potential avenue of disease development and prevention [21]. While a healthy relationship between gut microbiome organisms and the host can be beneficial, dysbiotic relationships can be detrimental to the host and lead to adverse health outcomes [30,31,32]. This is of particular interest among the elderly population, as the changes that occur in the gut microbiome throughout the aging process have been linked with unhealthy aging [33], which includes the development of chronic diseases [34]. Ensuring individuals maintain their microbial diversity may be beneficial in delaying or preventing the onset of some diseases [35]. Aging, lifestyle, diet and host-immune system functionality have been identified as factors that can affect the gut microbiome composition, altering its interaction with the host [36]. 

There is an overlap between the health benefits of anthocyanins and an optimally functioning microbiome. This review will examine four health outcomes prevalent in the older adult population-CVD, various forms of cancer, neurodegenerative disorders and aging-associated bone loss-that have shown to be influenced by anthocyanins and gut microbiome composition. Collectively, these diseases cost the United States hundreds of billions of dollars each year and are responsible for millions of hospitalizations and deaths throughout the country [37,38,39]. The aim of this review is to examine current research on anthocyanins not only as an independent influencer of health, but also their role in modulating the gut microbiota to work synergistically to improve health and function and as potentially cheaper alternatives or adjuvants to traditional methods of chronic disease treatment and prevention. 

PubMed was used to identify articles for this review. To determine what health benefits researchers have studied related to anthocyanins and the microbiome, the terms “anthocyanins and health”, “microbiome and health” and “microbiome, anthocyanins and health” were searched in the database. The following words were added to find specific evidence as to how anthocyanins and the gut microbiome effect health: aging, cardiometabolic effects, atherosclerosis, HDL cholesterol, blood pressure, cardiovascular disease, cancer, colon cancer, breast cancer, neuroprotection, neurodegenerative diseases, Alzheimer’s disease, bone and bone loss. The term “gut microbiota and anthocyanins” was used to examine their interaction. All articles included in this review were published prior to October 2020. 

## 2. Bioavailability of Anthocyanins

In the case of anthocyanins, bioavailability can be defined as the fraction of anthocyanins that are absorbed and utilized by the body [40]. Anthocyanins have been suggested to be minimally absorbed by GI tract through the stomach and the small intestine [8], via active transporters at those sites and transported to other tissues in the body like the kidney and liver [41]. After absorption, anthocyanins can also have an effect on structures in the brain by being transported across the blood brain barrier and localizing in various regions [42]. A large amount of the anthocyanins, however, bypass the small intestine and make their way to the colon to be further digested through both microbiome-mediated hydrolysis and fermentation processes [17]. The resulting colonic metabolites contribute to the biological effect attributed to anthocyanins [18] and are transported to the liver to be further metabolized [8,17]. There are two major methods of flavonoid excretion: urinary excretion via the kidney and bile excretion [8], with some of the catabolized flavonoid compounds excreted through the bile reabsorbed by the small intestine [17].

The bioavailability of anthocyanins in their native form has been suggested to be low at 1–2% [41]. However, newly identified metabolites of anthocyanins indicate that their bioavailability may be larger than previously suggested [43], with evidence from Czank et al. showing a 12.38% bioavailability of cyanidin-3-glucoside (C3G) when using an isotopically labeled tracer of the anthocyanin [44]. Anthocyanins and their catabolites are subjected to phase 2 enzymatic metabolism, leaving their methylated, glucuronidated and sulphated forms to be transported and utilized by the body [41]. These forms are present in the body at a higher concentration than the native structure and have been suggested as the compounds responsible for the health benefits associated with anthocyanin consumption [18]. 

## 3. Anthocyanin Effect on Health

Anthocyanins have been suggested to be effective against adverse health outcomes associated with aging, namely CVD, certain forms of cancer, neurodegenerative disorders and aging-associated bone loss. While many of the studies discussed in this section examine the role of dietary sources of anthocyanins at a particular point in time rather than throughout the aging process, they can still provide insight into the potential ways that anthocyanins could be utilized in the prevention and treatment of the health outcomes. It is important to note that many of the studies administer a dietary intervention to see the effect of the source’s total anthocyanins, rather than isolate a single form of anthocyanin. Dietary sources of anthocyanins contain many different anthocyanins and the preparation of the source will have an impact on the anthocyanin profile [45]. For example, Prior et al. outlined the anthocyanin profile of lowbush blueberries and isolated 19 different anthocyanins in the fruit, with contributions ranging from 1.1% to 14.4% [46]. Additionally, the harvest season and genotype can impact the anthocyanin profile [47]. This makes it difficult to determine the role of specific parent anthocyanins in health. Future studies need to be conducted to further examine the potential of dietary sources of anthocyanins in the prevention or treatment of adverse health outcomes in the aging population, since evidence suggests anthocyanin-rich dietary sources may be a useful intervention in four of the most prominent health issues of this population. A summary table (Table 1) outlining common dietary sources of anthocyanins, the prominent parent anthocyanins in those sources and their documented role in disease prevention has been included.

### 3.1. Cardiovascular Disease

Adverse cardiac events have been linked to reduced blood flow ability; two conditions that capture this criterion are hypertension and atherosclerosis [13]. The effect of anthocyanins at managing certain risk factors for CVD has been studied, including cholesterol levels [49,63] and blood pressure [64]. 

High density lipoprotein cholesterol (HDL-C) has an anti-atherosclerotic effect, with increased levels associated with decreased risk of atherosclerosis [65,66]. Elevated levels of low-density lipoprotein cholesterol (LDL-C) are associated with increased risk of atherosclerosis development [66]. In their placebo-controlled crossover study, Hassellund et al. found that a four-week anthocyanin intervention significantly increased HDL-C levels in prehypertensive men. The intervention capsule contained a total of 80 mg of 17 different anthocyanins from blueberries and blackcurrants, with the majority of the capsule containing cyanidin 3-O-*β*-glucosides and delphinidin 3-O-*β*-glucosides [49]. Interestingly, evidence from Xie et al. displayed no difference in plasma HDL-C levels, while LDL-C levels significantly decreased in the aronia berry extract treatment group [63]. Anthocyanins had no significant effect on biomarkers for inflammation and oxidative stress, which influence CVD risks [49,63] and therefore the underlying mechanisms for its action on modulating lipids is unclear. 

In one study, anthocyanins reduced blood pressure by a similar magnitude as captopril, an angiotensin-converting enzyme (ACE) inhibitor used to treat high blood pressure [64]. Herrera-Arellano et al. found no significant difference in the effectiveness of a daily 9.6 mg anthocyanin infusion prepared from Hibiscus sabdariffa, which contains the anthocyanins delphinidin and cyanidin as well as their glycoside forms, compared to 25 mg captopril to decrease blood pressure in patients experiencing mild to moderate hypertension, with patients receiving the anthocyanin treatment having their average systolic and diastolic blood pressure reduced from 139.05 to 123.73 mm Hg and from 90.81 to 79.52 mm Hg, respectively [64]; the reduced levels are below the Centers for Disease Control and Prevention’s definition of hypertension [67]. 

Anthocyanin supplementation in the aging population may be relevant in the treatment and prevention of CVD, as evidence suggests anthocyanins may have a positive effect on reversing hypertension and atherosclerosis development, subsequently decreasing overall CVD risk. 

### 3.2. Cancer

Increased cell proliferation and decreased apoptosis are factors that lead to the development and progression of cancer [68]; anthocyanins may have an effect on these processes [9,13,16,69,70,71,72,73,74,75]. The mechanism of action is unconfirmed, with multiple mechanisms likely working in tandem [71]. Seeram et al. examined this association in vitro using human oral, breast, colon and prostate tumor cell lines and blackberry, black raspberry, blueberry, cranberry, red raspberry and strawberry extracts at concentrations ranging from 25 to 200 μg/mL. All the cell lines responded in a similar manner, in which there was an increased inhibition of cell growth as the concentration of berry extract increased [9]. Anthocyanins may be particularly effective against colorectal cancer [9,71,72,73,74,75], with evidence from a meta-analysis by Wang et al. suggesting a significant inverse relationship between total anthocyanin consumption and risk of colorectal cancer development [74]. Anthocyanins derived from the six berry species mentioned prior and the grumixama fruit may be particularly effective against breast cancer [9,76]. Not all cancers respond in the same way to anthocyanins. In their meta-analysis, Yang et al. concluded there was no association between various forms of anthocyanins and the risk of developing gastric cancer [77], indicating that anthocyanins may only be effective against certain forms of cancer. 

From 2011–2015, the rate of incidence of lung, prostate, breast and colorectal cancer were highest among older adults and from 2012–2016 the same forms of cancer were responsible for the highest mortality rates among the same population [78]. Of these most prevalent cancers among older adults, anthocyanins were found to be potentially effective against breast, colon and prostate cancers. More research needs to be completed to better understand the mechanism of anthocyanins as it relates to cancer development in aging and treatment, as well as outline the specific of cancers that anthocyanins may be the most useful. 

### 3.3. Neurodegenerative Disorder 

Consumption of anthocyanin rich foods, especially foods containing cyanidin, may affect neuronal apoptosis and death as well as learning and memory impairment, which are processes that can occur with aging [79]. Factors that contribute to disease progression include oxidative and nitrosative stress, excitotoxicity and dysregulation of calcium homeostasis, increased inflammation in the central nervous system (CNS) and death of specific neuronal populations [80]. Key mechanisms that have been suggested as ways anthocyanins exhibit neuroprotective properties are suppression of oxidative stress and neuroinflammation [79,81]. In their review Zhang et al. also identified four additional mechanism that may mediate the effects anthocyanins have on the CNS: suppression of c-Jun N-terminal kinase activation, amelioration of cellular degeneration, activation of brain-derived neurotrophic factor signaling and restoring Ca^2+^ and Zn^2+^ homeostasis [79]. 

Min et al. examined the neuroprotective role of the prominent anthocyanin cyanidin-3-O-glucoside (C3OG) in mice. Researchers subjected mice to permanent middle cerebral artery occlusion and used C3OG purified from tart cherries as the treatment. They found that C3OG significantly reduced cerebral infarct size, strengthened neurological functional outcome and decreased levels of superoxide in the brain [81]. 

Using an APP/PS1 mouse model of AD and a dietary supplementation of anthocyanins extracted from Korean black beans, Ali et al. found that the antioxidative effects of anthocyanins can prevent neurodegeneration through the P13K/Akt/Nrf2 pathway, improve memory related pre- and postsynaptic markers and improve cognitive functions, leading researchers to conclude that anthocyanins may be utilized as a dietary supplement in the aging population to prevent neurological disorders associated with aging [82]. Vepsäläinen et al. also used a APP/PS1 mouse model of AD to evaluate the potential role of anthocyanins in the prevention of AD. Investigators compared the efficacy of three diets- a standard diet, a standard diet supplemented with purified bilberry anthocyanin powder and a standard diet supplemented with purified blackcurrant anthocyanin powder- on neuroprotection and concluded that both bilberry and blackcurrant extracts could be viable in this regard through the beneficial effect they exert on amyloid precursor protein and *β*-amyloid metabolism, which have been identified as pathogenic features of AD. The anthocyanin rich powders also had a positive effect on behavioral abnormalities associated with AD [55]. 

Anthocyanins from the diet may have a role in the prevention of neurodegeneration in the aging population. Their anti-oxidative properties have been suggested to be a factor in their potential role and their ability to cross the blood brain barrier has been attributed to their suggested beneficial effects on structures in the CNS. More research, especially in human subjects, needs to be completed in order to evaluate the efficacy of using anthocyanin rich foods for neuroprotection in the aging population. 

### 3.4. Aging-Associated Bone Loss

Studies have indicated a possible beneficial relationship between anthocyanins and bone loss [14,56,83,84,85,86,87], as bone loss has been linked to increased oxidative stress and inflammation [56]. In aging, there is an acceleration in the rate of bone mass loss as well as an overall weakening of the bone [88]. This can lead to osteoporosis development, with osteoporotic individuals at an increased risk of fractures [38]. This may be of particular interest for women, as their decline in estrogen levels during menopause is a risk factor for osteoporosis development [89]. 

Sakaki et al. examined this relationship using a mouse model of age-related bone loss; blackcurrant extract (BCE) was the anthocyanin rich supplementation in the chow diet, which was compared to a standard chow diet. Trabecular bone mass increased by 43.2% in BCE-supplemented young mice, while bone mass was not significantly altered in BCE-supplemented aged mice. These results suggest BCE supplementation can prevent age-related bone loss, but the benefits may only be beneficial when the supplementation happens prior to sufficient aging [56]. 

Bilberry extract, which contains 15 different anthocyanins, was found to not have an effect on bone metabolism in ovariectomized (OVX) rats [90]. However, this model of bone loss imitates the process of postmenopausal bone loss which is similar but not definitively analogous to age-related bone loss [91]; this difference could explain the discrepancies within the results. Interestingly, Zheng et al. found that BCE supplementation reduced ovariectomy-induced bone loss in mice [14]. Nagaoka et al. had similar conclusions when they used supplemented mice with maqui berry (MB) extract in an OVX model. MB is rich in the anthocyanin delphinidin, which has been shown to inhibit osteoclast differentiation, a bone-resorbing cell, while promoting osteoblast differentiation, a bone-synthesizing cell [84]. Moriwaki et al. also found that delphinidin suppresses the formation of osteoclasts, while cyanidin and peonidin did not have as strong of an effect on osteoclasts [92]. 

Anthocyanins may be beneficial in preventing aging-associated bone loss, especially when consumed prior to excessive losses. However, not all studies have shown this benefit, indicating there may be differences in anthocyanin effectiveness against age related bone loss and postmenopausal bone loss. The dietary source of the anthocyanin may also be of importance, as well as the specific anthocyanin compounds.

## 4. Anthocyanins, Microbiome Composition and Aging Related Health Effects

The microbiome changes with age, which has led to microbiome dysbiosis to be a suggested biomarker of aging [93]. Aging has been suggested to affect microbiome composition [94] through physiological changes, like immunosenescence and inflammaging [95], or changes in dietary patterns [96]. The exact composition of the microorganisms residing in the gut varies [23]. However, the presence of a core microbiota has been described [97,98] that encompass prevalent families of bacteria [98], including the Lachnospiraceae, Ruminococcaeae and Bacteroidaceae families [33]. Older adults often experience problems with frailty, which has been associated with poor microbial diversity. The proportion of Bacteroidetes in frailer elderly individuals tends to be higher than younger individuals [94]. Claesson et al. concluded there was a change in core microbial diversity in elderly subjects because they had a distinct core microbiome. Specifically, researchers found that there was a greater proportion of the Bacteroides species as well as an increase in the Clostridium species [97]. Biagi et al. had similar findings and suggested levels of the core microbiota families listed above decrease with age [98].

There may be a relationship between consumption of anthocyanin-rich foods and gut microbiome composition, which includes increasing the overall quantity [99] as well as increasing the growth of specific microbial substances [19] (Table 2). Anthocyanin’s effect on microbial diversity in the gut could be particularly beneficial in reducing the risk of developing CVD [100] and colorectal cancer [32] since risk has been linked to a lack of microbiome diversity [15,100]. 

### 4.1. Anti-Inflammatory and Anti-Oxidative Effects of Parent Anthocyanins, Mediated by the Microbiome

Few studies have examined the role of both the microbiome and specific anthocyanins in health. Generally, these studies have focused on anthocyanins ability to alter the gut microbiome, ultimately reducing inflammation status [101,102] or oxidative stress [103]. Evidence from the abundant anthocyanin cyanidin-3-glucoside (C3G) depicts a potential mechanism on the maintenance of gut integrity, which subsequently provides health benefits. C3G catabolism in the gut microbiome results in the production of phenolic compounds, including protocatechuic acid, vanillic acid, phloroglucinaldehyde and ferulic acid, which have an effect on oxidative stress and inflammation on the gut [11]. Evidence suggests that these metabolites can activate Nrf2 [11], which manages antioxidant enzymes and proteins [104]. They may also responsible for reducing inflammation in the gut by affecting the TAK1-mediated MAKP and SphK/S1P mediated NF-κB pathways [11]. Thus, C3G and its metabolites play a role in reducing inflammation and oxidative stress in the gut, which helps to provide optimal conditions for metabolism to occur. The anthocyanin malvidin-3-glucoside (M3G) may also be a potential mechanism on the maintenance of gut integrity. In a batch-culture fermentation system, which modeled the human distal large intestine, Hidalgo et al. concluded that not only a mixture of anthocyanins could enhance *Bifidobacterium* and *Lactobacullus-Enterococcus* growth, but also that M3G could individually have the same effect [105].

**Table 2:** Significant gut microbiome alterations following consumption of anthocyanin rich foods ^a^.

Study	Subjects/Animals	Intervention	Observed Changes to Microbiome Composition
Lee et al. (2018) [99]	Male Wistar rats	LF (10% fat), HF (45% fat), or HF with 10% by weight blueberry powder diets for 8 weeks	Phylum level changes: decrease in Firmicutes, Bacteriodetes, increase in Proteobacteria, Fusobacteria compared to HF and LF groups SCFA changes: elevated serum levels of acetate compared to HF and LF groups, elevated levels of propionate compared to LF group, lower serum levels of butyrate compared to LF group 3-fold increase in SCFA-target receptor expression (Gpr43) compared to the LF group
Pan et al. (2017) [19]	Male F-344 rats	Control diet, control diet and 5% whole black raspberry powder, control diet and 0.2% black raspberry anthocyanins, or control diet and 2.25% of residue fraction for 6 weeks	Control diet: increase in *Asaccharobacter* and decrease in *Clostridium, Acetanaerobacterium* 5% whole black raspberry powder: increase in *Anaerostripes, Ruminococcus*, *Akkermansia*, *Coprobacillus,* decrease in *Acetivibrio* 0.2% black raspberry anthocyanins: increase in *Anaerovorax, Dorea*, decrease in *Bifidobacterium*, *Lactobacillus* 2.25% residue fraction: increase of *Anaerotruncus, Coprobacillus*, *Desulfovibrio*, *Victivallis*, *Mucispirillum*, decrease in *Streptococcus*, *Turicibacter, Acetivibrio*
Anhê et al. (2015) [106]	Male C57BI/6 J mice	Standard chow, HFHS diet, HFHS diet and 200 mg/kg body weight cranberry extract for 8 weeks	Cranberry extract group was associated with 30% increase in relative abundance of *Akkermansia*
Gu et al. (2019) [107]	Male C57BL/6 x FVB F1 mice	Control diet or 10% *w*/*w* black raspberry diet for 6 weeks	Colon microbial α-diversity significantly greater in black raspberry fed mice Phylum level changes: decrease in Firmicutes, increase in Bacteroidetes black raspberry fed mice Genus level changes: decrease in *Clostridium*, *Lactobacillus*, increase in *Barnesiella* in black raspberry fed mice
Liu et al. (2017) [108]	Male C57BL/6 mice	Standard diet, HF diet, or HF diet and 300 mg/kg body weight GSPE for 7 weeks	GSPE treated mice had a significantly different *β*-diversity Phylum level changes: decrease in Firmicutes, increase in Proteobacteria in GSPE group Family level changes: increase in *Lachnospiraceae*, *Peptostreptoccaceae*, *Erysipelotrichaceae*, *Veillonellaceae*, *Prevotellaceae* in GSPE group Genus level changes: increase in *Prevotella*, *Clostridium XIVa, Escherichia/Shigella*, *Blautia*, *Flavonifractor*, *Arthrobacter*, decrease in *Lactococcus*, *Bacteroides*, *Roseburia* in GSPE group
Molan et al. (2014) [109]	Healthy men and women aged 20–60 years	672 mg of BCE: First Leaf (BCE powder, lactoferrin, lutein) or Class Anthomix 30 (BCE powder) for 2 weeks	Both treatments decreased *β*—glucuronidase enzyme activity and levels of *Bacteroides*, Clostridia, increased levels of bifidobacteria, *Lactobacillus*
Petersen et al. (2019) [110]	Control (*db/+)* and diabetic mice (*db/db)*	Standard diet or diet supplemented with 2.35% freeze dried strawberry for 10 weeks	α-diversity indices *β*-diversity were different among treatment groups and microbial composition was significantly influenced by genotype (*db/db)* and strawberry consumption Genus level changes: decrease in *Bifidobacterium* and increase in *Bacteroides* in mice fed diet containing strawberries
Guglielmetti et al. (2013) [111]	Healthy men with at least one CVD risk factor	250 mL of wild blueberry drink for 6 weeks (crossover design)	Blueberry drink consumption selectively increased bifidobacteria
Neyrinck et al. (2013) [112]	Balb/c mice	Control diet, HF diet, HF diet and pomegranate peel extract (0.2% in water)	Significant increase in *Bifidobacterium* spp., nearly significant increase in *Bacteroides-Prevotella* spp.
Mayta-Apaza et al. (2018) [113]	*In vitro* digestions; healthy men and women divided into low (LB) or high (LB) *Bacteroides* groups	In vitro: 5 mL of tart cherriesHuman participants: 8 ounces of tart cherry juice for 5 days	In vitro: large increase in *Bacteroides*, *Collinsella*, moderate increase in Firmicutes, *Enterobacteriaceae*, *Bilophila* HB human participants: decease in, *Bacteroides*, increase in various Firmicutes (*Ruminococcus*, *Lachnospiraceae*, *Clostridium* and *Clostridium XI*, *Dialister*, *Coprococcus*, *Lactobacillus*, *Streptococcus)*, some Actinobacteria, *Collinsella* LB human participants: generally opposite changes (increase in *Bacteroides*, *Bifidobacterium*, decrease in Firmicutes)
Jakobsdottir et al. (2013) [114]	Male Wistar rats	Standard diet with freeze dried blackcurrant, blackberry, or raspberry, either with or without HEAL19 for 5 days	Total cecal pool of SCFAs, acetic acid, propionic acid and butyric acid were higher in blackcurrant group In proximal and distal colon, blackcurrant generally yielded higher levels of SCFAs. Microbial diversity of rats fed raspberries was higher
Marques et al. (2018) [115]	Male Wistar rats	Standard diet, standard diet and BE, HF diet, HF diet and BE for 17 weeks	Genus level changes of standard diet and BE: increase of *Pseudoflavonifactor* Genus level changes in both BE groups: increase of *Oscillobactor* Genus level changes in HF and BE group: decrease in *Rumminococcus* and increase in *Sporobacter* compared to HF group

^a^ HF: high fat; LF: low fat; SCFA; short chain fatty acid; HFHS: high fat/high sucrose; GSPE: grape seed proanthocyanindin extract; BCE: blackcurrant extract; CVD: cardiovascular disease; HEAL19: *Lactobacillus plantarum* HEAL19; BE: anthocyanin rich blackberry extract; LB: low *Bacteroides*; HB: high *Bacteroides*.

### 4.2. Cardioprotective Effects of Anthocyanin Containing Foods, Mediated by the Microbiome

Cholesterol transport may be affected by both anthocyanins and gut microbiota. Wang et al. concluded that the anthocyanin derived metabolite protocatechuic acid promotes macrophage cholesterol efflux by downregulating miR-10b expression, which represses ATP-binding cassette transporter A1 and ATP-binding cassette transporter G1 [116]. Gallic acid (GA), another anthocyanin metabolite, may have two pathways at which it can be beneficial to health. It has been shown to reduce levels of the potentially pathogenic *Clostridium histolyticum* without impacting the growth of beneficial bacteria [105]. GA also has demonstrated a positive effect on CVD risk, as evidence suggests it can act as an ACE inhibitor yielding results similar to the effects of captopril, a medication used to treat high blood pressure [117]. 

Pan et al. used F-344 rats to examine the relationship between anthocyanins and gut microbiome composition, giving the experimental groups one of three black raspberry treatments. Their results showed that not only did the composition of the microbiome change, but also highlighted the specific beneficial microbial species that increased. Examples include *Akkermansia* and *Desulfovibrio*-which have anti-inflammatory properties-and *Anaerostipe* [19]-which is a butyrate producing bacteria that has demonstrated prevention of certain diseases [118]. Anhê et al. used an in vivo mice model to show that anthocyanin rich cranberry extract may also be effective at increasing the *Akkermansia* species in the gut [106]. Schneeberger et al. demonstrated that increasing the specific species *Akkermansia mucinphila* may exhibit some cardioprotective effects by modulating factors related to obesity, like protecting against body weight gain and decreasing inflammation in adipose tissue, in male age matched mice [119], indicating the alterations anthocyanin rich foods make in the gut microbiome may have cardioprotective effects. 

Gu et al. also examined the effect of anthocyanins in black raspberries on the gut microbiome composition. Male mice supplemented with a 10% *w*/*w* freeze dried black raspberry powder showed greater microbiome diversity. *Clostridium*, which contains many pathogenic species, significantly decreased, while *Barnesiella*, a recently discovered genus with potentially beneficial effects, increased [107]. Another study that found anthocyanin containing foods may modulate *Clostridium* was conducted using grape seed proanthocyanidin extract (GSPE). After supplementing mice with 300 mg/kg body weight of GSPE for seven weeks, levels of *Clostridium XVIa, Roseburia* and *Prevotella* increased [108]. 

While generally low *Clostridium* levels are thought to be beneficial to the elderly population, *Clostridium XVIa* does not have prominent toxins and virulence factors associated with the pathogenic effects of the genus [120] and may inhibit macrophage infiltration in adipose and hepatic tissues [121]. The increase in *Roseburia* levels may also be of interest with regards to atherosclerosis risk. Karlsson et al. sequenced the gut metagenomes of individuals with symptomatic atherosclerotic plaque and compared them to gender and age matched controls. Among other microbiome alterations, investigators found elevated levels of *Roseburia* and *Eubacterium* in the healthy controls and elevated levels of *Collinsella* in the patients [122]. This indicates the gut metagenome may be related to symptomatic atherosclerosis development [122] and dietary sources of anthocyanins may be able to aid in providing the desired *Roseburia* levels [108].

Molan et al. also found a beneficial modification of the gut microbiota that may protect against the shift in core gut microbiota in older adults through the administration of an anthocyanin rich powder derived from blackcurrants. In their experimental human study, researchers saw a decrease in both *Clostridium* and *Bacteroides,* which have both been documented as being contributing factors to the microbial shift in elderly individuals. Further modifications include an increase in the beneficial microbial groups *Lactobacillus* and *Bifidobacterium* [109]. Those microbial groups have been associated with the following: pathogen suppression in the gut [123], colon cancer prevention [124], synthesis of vitamins and strengthening the immune system [109]. However, Yamashiro et al. have found that elevated counts of the species *Lactobacillus ruminus* was associated with ischemic stroke inflammation through increased serum interlukin-6 [125]. Therefore, the specific species of *Lactobacillus* bacteria that anthocyanins increase may be of importance to the elderly population since aging is the primary non-modifiable risk factor for ischemic stroke [126]. With regards to *Bifidobacterium,* evidence suggests anthocyanin containing foods may be particularly effective at increasing *Bifidobacterium* levels in the gut, as both human and animal studies that supplemented with anthocyanin-rich blackcurrants [109], strawberries [110], blueberries [111], pomegranates [112], tart cherries [113] and jussara fruit [127] have documented this effect. 

Blackcurrants have had other demonstrated effects on the gut microbiome. Cao et al. studied the effects of BCE on the gut microbiome in female mice. When supplemented with BCE for four months, the relative levels of Firmicutes, Bacteroidetes, Cyanobacteria, Proteobacteria and Tenericutes were higher in young mice compared to aged mice. Another finding of this study suggested the gut microbiome of young and aged mice responded differently to BCE supplementation, indicating the timing of supplementation may be important for maximal effect in the aging population. Finally, the Firmicutes/*Bacteroides* ratio was affected [128]. An increase of this ratio has been shown to be associated with hypertension [129] and to be a negative predictor of bone volume [130]. The Firmicutes/*Bacteroides* increased with aging but was reduced by BCE treatment [128], indicating the effect of anthocyanins in blackcurrants may be through modification of the composition of the gut microbiome. The increase in Tenericutes may be relevant in regulating CVD risk factors related to the aging population [131,132]. In the Metabolic Syndrome in Men study, Ahmadmehrabi et al. saw an abundance of Tenericutes, as well as *Christensenellaceae, Methanobrevibacter* and *Peptococcaceae* were associated with reduced triglyceride levels; Tenericutes and *Christensenellaceae* were also directly associated with a reduced body mass index and increased HDL-C levels [133].

One major pathway related to dybiosis associated with CVD risk is the SCFA pathways [133]. Macromolecules that enter the distal gut are fermented by colonic bacteria, producing SCFAs—the most abundant including acetic acid, propionic acid and butyric acid [133]—which will enter circulation [134] and act as an energy source in a metabolic cross-feeding process [133]. Anthocyanin containing foods may elevate SCFA levels. Jakobsdottir et al. found that rats fed diets supplemented with blackcurrants had higher production of SCFAs [114]. Olfactory receptor 78 (Olfr78) and G protein couple receptor 41 (Gpr41) are two sensory receptors for SCFAs which regulate blood pressure [134]. SCFAs produced by the gut microbiome may regulate blood pressure through the modulation of renin release via Olfr78 in the arteriole and by modulating peripheral resistance through Olfr78 and Gpr41 [134].

### 4.3. Neuroprotective Effects of Anthocyanin Containing Foods, Mediated by the Microbiome 

Anthocyanins attenuate neurodegenerative pathologies, possibly through their ability to modulate the gut microbiome. Marques et al. examined this potential link in rats being fed high fat diets, as high fat diets are thought to contribute to neuroinflammation and neurobehavioral changes in obesity through gut microbiome alterations. Researchers used four treatment groups (standard diet, standard diet and anthocyanin rich blackberry extract (BE), high fat diet and high fat diet and BE) and found that animals supplemented with BE experienced modification in their gut microbiomes, including the increase of *Pseudoflavonifractor* and *Oscillibacter.* They concluded that the modulations in the gut by BE were correlated with anti-neuroinflammatory properties through decreasing TCK-1 expression and that BE could impact CNS inflammation through altering tryptophan metabolism along the kynurenine pathway, thus increasing the production of the neuroprotective metabolite [115].

Evidence suggests foods containing anthocyanins may affect the composition of the gut microbiome by increasing the number of beneficial bacteria and decreasing the number of potentially pathogenic bacteria. Optimizing the gut microbiome through anthocyanin supplementation may be of interest for the older adult population, as both have been shown to have a beneficial effect on chronic diseases prevalent in that population. More studies need to be completed that examine the role of both anthocyanins and the gut microbiome in the prevention or treatment of diseases. There may be an overlap in their mechanism of action regarding specific chronic diseases affecting older adults, especially in the context of inflammation and oxidative stress. Examining the interaction between anthocyanins and the gut microbiota is crucial when attempting to understand their individual actions. 

## 5. Conclusions

The effect of anthocyanins and the gut microbiome on health have been studied, with evidence suggesting both factors play a role in the prevention and treatment of negative health outcomes significantly affecting the aging population including CVD, certain cancers, neurodegenerative disorders and aging-associated bone loss. The role of the gut microbiome to mediate the effects of anthocyanins is of interest because anthocyanin derived metabolites from the microbiome have been suggested to contribute to the positive biological effects attributed to anthocyanins. This could be especially true for the aging population because there has been a documented shift in the core microbiome of this population that has been linked to their frailty. While a healthy microbiome composition can be protective against disease, a dysbiotic relationship could prove to be detrimental to the host as it can lead to the development of negative health effects. The relationship between the gut microbiome and anthocyanins has been minimally explored, with some evidence suggesting the interaction between these components may be a mechanism of action for the health benefits seen. Many studies use a dietary intervention and examine the role of anthocyanin-rich foods containing many different anthocyanins, rather than the role of specific anthocyanins; it is difficult to determine how individual anthocyanins may affect health or how the gut microbiome may mediate the effects of individual anthocyanins. Based on the evidence from the literature, consuming anthocyanin-rich foods may be a potential avenue for disease prevention in the aging population, with minimal side effects compared to conventional methods. However, the timing of the anthocyanin consumption may be important, with some studies suggesting consuming anthocyanins early may have a protective effect later in life. More studies need to be completed that specifically examine both the microbiome and anthocyanins on managing health and preventing disease in the aging population. 

## Figures and Tables

**Table 1:** Common dietary sources of anthocyanins and their documented health outcomes related to cardiovascular disease, cancer, neurodegenerative disorder and aging-associated bone loss.

Dietary Source	Prominent Parent Anthocyanins	Suggested Health Outcomes ^a^
Blueberry	Malvidin [46,48], delphinidin, petunidin [46]	Increases HDL-C levels [49]; decreases LDL-C, total TG and adiponectin [50]; reduction of arterial stiffness and improvement of blood pressure [51]; inhibition of cancer cell growth [9]; improves aged rats spatial working memory task performance [52]
Blackberry	Cyanidin [48]	Inhibition of cancer cell growth [9,53]; protect against LDL-C oxidation and suppress cytokine induced MCP-1 secretion [53]
Blackcurrant	Delphinidin [48,54], cyanidin [54]	Increases HDL-C levels [49]; improves behavioral outcomes, APP processing and A*β* accumulation [55]; increases trabecular bone mass [56]; reduces ovariectomy-induced bone loss [14]
Red Raspberry	Cyanidin, pelargonidin [47]	Reduction in lipid accumulation [57]; inhibition of cancer cell growth [9]
Cranberry	Cyanidin, peonidin [46]	Improves total cholesterol ratio [58,59]; improves vascular function [60]; Inhibition of cell cancer growth [9]
Strawberry	Cyanidin, pelargonidin [61]	improves lipid profile by decreasing total cholesterol and LDL-C levels [62]; Inhibition of cancer cell growth [9]

^a^ HDL-C: high density lipoprotein cholesterol; LDL-C: low density lipoprotein cholesterol; TG: triglycerides; MCP-1: monocyte chemotactic protein 1; A*β*: *β*-amyloid; APP: amyloid precursor protein.

## Data Availability

No new data were created or analyzed in this study. Data sharing is not applicable to this article.
